# Investigation of Cell Mechanics and Migration on DDR2-Expressing Neuroblastoma Cell Line

**DOI:** 10.3390/life14101260

**Published:** 2024-10-02

**Authors:** Theadora Vessella, Esteban J. Rozen, Jason Shohet, Qi Wen, Hong Susan Zhou

**Affiliations:** 1 Department of Chemical Engineering, Worcester Polytechnic Institute, 100 Institute Rd., Worcester, MA 01609, USA; tvessella@wpi.edu; 2 Crnic Institute Bolder Branch, BioFrontiers Institute, University of Colorado Boulder, 3415 Colorado Avenue, Boulder, CO 80303, USA; 3 Department of Pediatrics, University of Massachusetts Medical School, 55 Lake Ave North, Worcester, MA 01655, USA; 4 Department of Physics, Worcester Polytechnic Institute, 100 Institute Rd., Worcester, MA 01609, USA

**Keywords:** cancer metastasis, DDR2, neuroblastoma

## Abstract

Neuroblastoma is a devastating disease accounting for ~15% of all childhood cancer deaths. Collagen content and fiber association within the tumor stroma influence tumor progression and metastasis. High expression levels of collagen receptor kinase, Discoidin domain receptor II (DDR2), are associated with the poor survival of neuroblastoma patients. Additionally, cancer cells generate and sustain mechanical forces within their environment as a part of their normal physiology. Despite this, evidence regarding whether collagen-activated DDR2 signaling dysregulates these migration forces is still elusive. To address these questions, a novel shRNA DDR2 knockdown neuroblastoma cell line (SH-SY5Y) was engineered to evaluate the consequence of DDR2 on cellular mechanics. Atomic force microscopy (AFM) and traction force microscopy (TFM) were utilized to unveil the biophysical altercations. DDR2 downregulation was found to significantly reduce proliferation, cell stiffness, and cellular elongation. Additionally, DDR2-downregulated cells had decreased traction forces when plated on collagen-coated elastic substrates. Together, these results highlight the important role that DDR2 has in reducing migration mechanics in neuroblastoma and suggest DDR2 may be a promising novel target for future therapies.

## 1. Introduction

Metastasis is a hallmark of aggressive cancer, and the metastatic cascade is characterized by migrating cancer cells interacting with their local microenvironment to sense biomechanical properties and adapt their migratory phenotype. Neuroblastoma (NB) is the most common extracranial solid tumor in childhood, with metastatic spread seen in half of the patients [1,2]. NB accounts for ~8% of all childhood cancers, but around 15% of pediatric cancer mortality [3]. Despite efforts in the clinic, 50–60% of high-risk patients will relapse within two years of a diagnosis [2]. The extracellular matrix has been shown to play critical roles in cancer initiation, maintenance, and aggressiveness [4,5], including neuroblastoma [6]. Therefore, a deeper understanding of how the extracellular matrix (ECM) regulates various cellular functions, such as cell spreading, migration, proliferation, and differentiation [7,8,9], is vital to improve treatments for NB.

Collagen is the major component of the extracellular matrix (ECM) in most mammalian tissues [10]. Cells bind to the collagen through adhesion receptors, and these receptors then activate actomyosin machinery that is capable of generating forces required for migration and delivering important signals for growth and survival [11]. In addition to integrins, the discoidin domain receptors (DDR), DDR1 and DDR2, are well-known collagen receptors. The DDRs are widely expressed in human and mouse tissues with distinct distributions; DDR1 is enriched in epithelial cells, and DDR2 is mainly expressed in mesenchymal cells [12]. Compared to DDR1, DDR2 has a greater specificity for fibril-forming collagen [13,14,15]. While DDR2 plays an important role in normal development by regulating cell proliferation and ECM matrix remodeling [16,17,18,19], an elevated expression of DDR2 has been shown to correlate with increased metastasis in various cancers [19,20,21,22,23].

During metastasis, cancer cells detach from the primary tumor and migrate through the ECM to invade the surrounding tissues [24]. Knocking down DDR2 has resulted in decreased cell migration [22,25,26,27,28,29] in various cell types. Cell migration is regulated by multiple factors including cell–ECM adhesion, cellular contractility, and cell mechanics. However, the mechanisms by which DDR2 modulates neuroblastoma cell mechanics and cell migration are still unknown.

In the current work, we studied the cell mechanics and migration of a neuroblastoma cell line SH-SY5Y. This cell line has been extensively used as a model to study cancer progression [30]. To gain insights into the role of DDR2 and collagen binding on neuroblastoma cellular mechanics, tetracycline inducible-shDDR2 (shDDR2) was stably transfected into the SH-SY5Y cells. The utilization and engineering of shDDR2 allows for precise control of DDR2 downregulation within these cells. Additionally, tet-inducible systems exhibit low basal expression in the absence of doxycycline, resulting in minimal interference with normal cellular processes. Together, this shDDR2 cell line allows for a precisely controlled environment to investigate the consequences of the gene manipulation under conditions that mimic disease states. Novel biophysical methods were employed to elucidate the mechanical changes from DDR2-downregulated neuroblastoma cells. To investigate cell microrheological properties, atomic force microscopy was utilized, allowing us to extract single cell stiffness measurements. Additionally, it is imperative to unveil the traction forces that shDDR2 cells exhibit in biologically relevant elastic substrates. To mimic the in vivo environment, neuroblastoma cells were plated onto soft collagen-coated elastic substrates. This allowed us to conclude changes to the shDDR2 traction forces in a biologically relevant way. To the best of our knowledge, this is the first study to unveil cellular migration mechanics on SH-SY5Y cells utilizing the novel shDDR2 cell line. Through these techniques, our results suggested increased traction forces, cell elongation, and cell stiffness were key components to mediate neuroblastoma cellular mechanics. We confirmed that on-target inhibition of DDR2 decreased its specificity for collagen, particularly regarding cellular traction forces and traction stress. DDR2 is a strong prognostic marker of poor survival in various cancers and adult tumors [31]. This work defines a novel role for this mesenchymal marker in regulating the mechanisms of neuroblastoma migration mechanics, a widely understudied topic poised to illuminate potential innovations for the treatment of pediatric cancer.

## 2. Materials and Methods

### 2.1. Cell Culture

Human neuroblastoma cell line SH-SY5Y stably transduced with the Tet-pLKO-puro lentiviral vector expressing either a control non-targeting shRNA or shDDR2 were kindly provided by Dr. Jason Shohet (UMass Chan Medical School). Cells were cultured in Dulbecco’s modified Eagle’s medium (DMEM) supplemented with 10% fetal bovine serum (Gibco, Grand Island, NY, USA), 2 mM glutamine (Gibco), and antibiotics (penicillin and streptomycin) (Gibco, Grand Island, NY, USA).

### 2.2. Lentivirus Preparation and Infection

HEK-293T cells were maintained at 37 °C in Dulbecco’s modified Eagle’s medium (DMEM), supplemented with 10% FCS and antibiotics (100 units/mL penicillin and 100 μg/mL streptomycin). Cells were transfected with pVSV-G [32] and pCMV∆R8.91 [33], together with the pLKO.1-puro non-targeting vector (Sigma Mission clone SHC016; ‘shCTRL’, Livonia, MI, USA) or the Tet-pLKO.1-shRNA vector (Sigma Mission TRCN0000001418; ‘shDDR2’) using Lipofectamine™ 2000 reagent (Thermo Fisher Scientific, Waltham, MA, USA) as recommended by the manufacturer and following the recommendations of the RNAi Consortium (TRC) laboratory protocols with slight modifications. Twelve hours after transfection, the medium was replaced by DMEM supplemented with 30% FCS and antibiotics. Cell supernatants were harvested every 24 h, replaced with fresh medium, and stored at 4 °C until collection of the last harvest (72 h). At this point, the consecutive harvests were pooled, filtered through 0.45 mm filters and split in 3–5 mL aliquots, which were stored at −80 °C. SH-SY5Y cells were infected with shCTRL or shDDR2 lentiviral particles by adding a 1:1 mix of medium:viral supernatant for 24–48 h. Puromycin selection (2 μg/mL) was applied for 2–3 days and always compared to non-transduced control cells, which generally died within the first 24 h.

### 2.3. Western Blot

Western blot analysis was conducted using a standard protocol [34]. Briefly, cells grown to a 60–80% confluency were lysed in radioimmunoprecipitation assay (RIPA) lysis buffer (Prometheus Protein Biology Products #18-416) supplemented with Protease and Phosphatase Inhibitor Cocktails (Pierce, Thermo Scientific A32955 and A32957). Lysates were sonicated on ice, centrifuged at 15,000× *g* at 4 °C for 20 min, and the soluble protein fraction was collected. Protein extracts were quantified using a Pierce BCA Protein Assay Kit (Thermo Scientific #23227). A total of 30–50 μg of protein (see Supplementary File S2 for protein quantification data) were separated via SDS-PAGE using Novex™ WedgeWell™ 4–20%, Tris-Glycine Mini Protein Gels (Invitrogen, Carlsbad, CA, USA; Thermo Scientific, Waltham, MA, USA, XP04202BOX) and blotted onto a PVDF membrane using an iBlot transfer system and transfer stacks (Invitrogen, Thermo Scientific IB401001). Proteins were detected using SuperSignal™ West Pico PLUS Chemiluminescent Substrate (Thermo Scientific 34580). A ChemiDoc MP Imaging System (Bio-Rad, Hercules, CA, USA) was used for chemiluminescent detection and analysis. Primary antibodies were: from Cell Signaling Technology DDR2 (#12133); from MilliporeSigma (Darmstadt, Germany) Anti-GAPDH antibody (MAB374); and from R & D Systems human phopho-DDR1/DDR2 (Y796/Y740) antibody (MAB25382).

### 2.4. Lentivector Cell Maintenance

To maintain lentivector transfected cells, shCTRL cells are incubated at 37 °C and 5% $CO_{2}$ within tissue culture-treated petri dishes or T-75 flasks. Cells are maintained in DMEM with 10% fetal bovine serum (Gibco), 2 mM glutamine (Gibco), and antibiotics (penicillin and streptomycin) (Gibco). To maintain lentivector selection within shCTRL cells, puromycin is maintained within the culture medium at a concentration of 2 μg/mL. Medium and puromycin is refreshed every 2–3 days. Puromycin is used as a selection marker within the lentivector construct. Only cells that are integrated with the lentiviral vector construct (puromycin- resistant gene) will survive puromycin selection. To maintain lentivector selection within shDDR2 cells, puromycin is maintained within the culture medium at a concentration of 2 μg/mL, and doxycycline is maintained within the culture medium at a concentration of 1 μg/mL. Doxycycline allows control over the Tet-On system. Without doxycycline, the gene of interest remains inactive. Upon doxycycline addition, it binds to the transactivator protein, causing a conformational change that allows the transactivator protein to bind to the tet operator sequence. Upon this change, the transcription is activated. Doxycycline and puromycin are refreshed every 2–3 days. Cells harvested for experiments were used below passage 14.

### 2.5. Collagen-Coated Glass Slides

Coverslips measuring 25 mm × 25 mm were placed into a coverslip holder, submerged in 70% ethanol, and sonicated for 15 min. The cleaned coverslips were then rinsed with DPBS and incubated in 0.1 mg/mL of Rat Tail Collagen I solution (Corning, NY, USA) for 1 h at 37 °C. Following the incubation, the coverslips were rinsed 3× with DPBS waiting 10 min per wash.

### 2.6. Cell Spreading Area Measurements

Individual cells were allowed to attach onto collagen glass substrate at 37 °C and 5% $CO_{2}$. After 30 min of incubation, single cells were imaged using Olympus IX3 inverted microscope equipped with 40 × 0.6 NA objective (Center Valley, PA, USA) using the phase contrast mode. Cell area, aspect ratio, and circularity were measured using free-hand tool and shape descriptors within ImageJ version 1.54a 05 (NIH). Area is defined as the area of the selection in square units. Aspect ratio is defined as the ratio of the particles fitting an ellipse as a ratio of the major axis divided by the minor axis. Circularity is defined as the deviation of the area of a circle, with a perfect circle taken as a circularity of 1.0. For studies containing dimethyl sulfoxide (DMSO) (Sigma, Livonia, MI, USA), DMSO was introduced to cells before plating onto collagen- coated glass substrates.

### 2.7. Kymograph

Cells were plated onto collagen glass slides and allowed to attach for 30 min at 37 °C and 5%. After 30 min, cells were imaged using Olympus IX3 inverted microscope equipped with a 40 × 0.6 NA objective using the phase contrast mode for 30 min (5 s intervals). Three cells were imaged per dish, for a total of 1.5 h of imaging. Time-lapse images were analyzed using the straight line tool in ImageJ (NIH) and the Multi Kymograph plug-in. The slope of positive and negative lines on the kymograph are taken as the length of protrusion and contraction, respectively. For more details, refer to Figure 4.

### 2.8. Atomic Force Microscopy

All measurements were performed utilizing an MFP-3D-BIO atomic force microscope (Asylum Research, Santa Barbara, CA, USA) and DNP-10 cantilevers (Bruker, Camarillo, CA, USA) with a nominal spring constant of 0.06 N/m. To ensure reliability of measurements, each cantilever was calibrated for spring constant (k).

Live cells were measured 24 h after seeding on the desired surfaces to ensure proper spreading, while preventing high confluency. Measurements were limited to isolated cells to reduce the influence of cell–cell communication. Phase contrast microscopy was used in unison with AFM to align the cantilever tip over desired measurement areas of the cell. Individual force curves were taken in three locations in the perinuclear region of each cell to guarantee that the thickness of the cell was significantly greater than the distance the cantilever indented into the cell. Each force curve was taken at a velocity of 2 µm/s and to a trigger point of 0.2~1 nN. A total of 10~16 cells were measured per dish. Measurements were taken no longer than 30 min after the dish was removed from the incubator to ensure cell viability.

Using a custom MATLAB code, cantilever deflection as a function of sample indentation depth was extracted from AFM force curves. Stiffness values were determined from the deflection-indentation curve using the Hertz model with a conical tip:

(1) $$E=kd\pi (1-v^{2})/(2{∆}^{2}tan\emptyset )$$

where *k* is the cantilever spring constant, *d* is the cantilever deflection, *ν* is the Poisson’s ratio value (using 0.5), Δ is the sample indentation depth, and *ϕ* is half the conical opening angle of the AFM tip [35]. To minimize the effects of nonlinear effects, force-indentation curves were fit to the Hertz model over the first 200~500 nm indentation depth.

### 2.9. Polyacrylamide Substrate Preparation

Polyacrylamide gel substrates were prepared through the polymerization of 8% acrylamide and 0.04% bis-acrylamide to achieve a stiffness of 2 kPa. This polymerization process was initiated by a solution containing 0.1% ammonium persulfate and 0.3% N,N,N′,N′-tetramethylethylenediamine. To enable protein cotating, 0.1 mg/mL of Collagen type I (Corning) or 10 μg/mL of Fibronectin (AdvancedBioMatrix, Carlsbad, CA, USA) was cross-linked to the PAA gel surface using sulfo-SANPAH. The gels were submerged under 1 mg/mL of sulfo-SANPAH (G-Biosciences, St. Louis, MO, USA) solution and placed 2 inches below an 8 W ultraviolet UV lamp (Hitachi F8T5—365nm, Tokyo, Japan) and irradiated for 15 min. The gels were then washed with HEPES buffer and soaked with 0.1 mg/mL rat-tail collagen type I or 10 µg/mL fibronectin for 12 h at 4 °C. After protein coating, the protein solution was aspirated, and gels were placed in culture medium and incubated for 30 min at 37 °C before cells were seeded on them.

For traction force microscopy, we followed an established protocol [36] to fabricate gel disks of 18 mm in diameter and approximately 100 μm in thickness. These gel disks were prepared with 0.1 μm of red fluorescent beads (Life Technologies, Carlsbad, CA, USA) embedded just beneath the top surface. Square glass coverslips measuring 25 mm × 25 mm were cleaned, treated with 1% 3-aminopropyl-trimethoxysilane solution for 10 min, and then coated with 0.5% glutaraldehyde. Round glass coverslips, 18 mm in diameter, were plasma cleaned and coated with a thin layer of fluorescent beads. A 25 μL mixture of acrylamide, bis-acrylamide, and initiators was applied between the glutaraldehyde-coated square coverslip and the beads-coated round coverslip, followed by polymerization at room temperature for 15 min. Subsequently, the round coverslip was gently removed, leaving the resulting gel disk firmly attached to the square coverslip, with the embedded beads positioned within 2 μm below the gel surface.

### 2.10. Cell Traction Force Measurements

Cell traction forces were measured using traction force microscopy [36]. Cells were cultured on PAA substrates for 24 h before subjected to traction force microscopy. For each cell selected for traction force microscopy, a fluorescence image of the substrate was recorded to capture the marker beads in the stressed state. In addition, a phase-contrast image was acquired to record the morphology of the cell. Trypsin (Invitrogen) was then applied to disrupt cell–substrate interactions and cause the cell to detach from the substrate. A final fluorescent image of the substrate was taken to capture the marker beads in the relaxed, unstressed state. Bead displacements were calculated from the two fluorescent images using a particle image velocimetry toolbox written in MATLAB [37]. Traction stress on the gel surface was calculated from the bead displacements using finite element analysis software (Ansys, Inc., Canonsburg, PA, USA). The magnitude of total traction force (F) was calculated by integrating the magnitude of traction stress over the cell area.

### 2.11. 2D Cell Migration Assay

Cells were cultured in full complete DMEM (Gibco) with the addition of HEPES to allow CO_2_ exchange for prolonged live-cell imaging. Individual cells were plated onto collagen-coated slides and incubated overnight. Following incubation, cells were imaged using an Olympus IX83 inverted microscopy equipped with a 10 × 0.3 NA objective using the phase contrast mode. Time-lapse images were acquired at intervals of Δt=5 min for 4 h in duration. Centroids of cells were analyzed from the time-lapse images using ImageJ. The mean square displacement (MSD) of each individual cell was then calculated from the cell trajectory using the MSDAnalyzer [38] for MATLAB. The MSD for each cell type was calculated from the average MSD of individual cells and was fit to the anomalous diffusion model

(2) $$MSD=b\tau^{\alpha }$$

using the MATLAB Curve Fitting Toolbox with τ representing the time interval and α representing the anomalous exponent. The α-value is an important index for directional persistence; it will be equal to 1 for cells exhibiting normal Brownian motion and 2 for cells that move in a perfectly straight manner. The average speed of each cell was calculated from the cell trajectory by averaging the displacement magnitudes between two consecutive frames divided by Δt.

### 2.12. Statistics

Prism 9.0 GraphPad software was used for graph generation and statistical analyses. The significance level was set to *p* < 0.05. The number of independent biological replicates, sample sizes analyzed, and statistical tests used are stated in the figure legends.

## 3. Results

### 3.1. shRNA-Mediated Knockdown of DDR2

**Figure 1:**
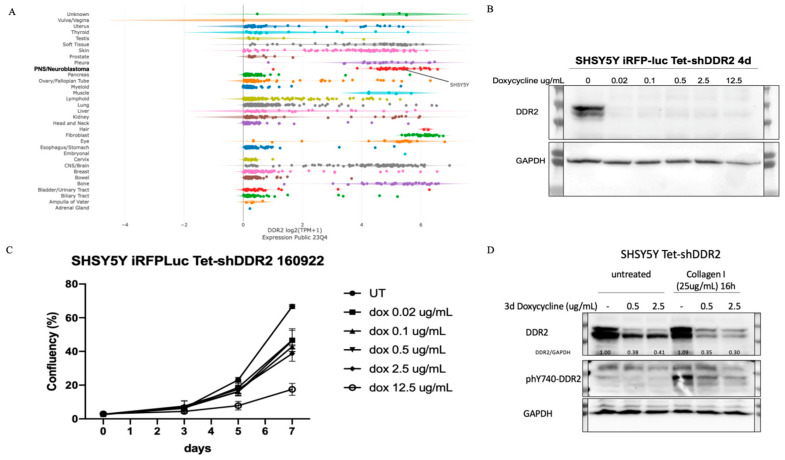
shRNA-mediated knockdown of DDR2. (**A**) DDR2 mRNA expression across cancer lines was ana-lyzed in the Dependency Map Portal (https://depmap.org/portal; Broad Institute, accessed on 15 February 2024). (**B**) Western blot of DDR2 expression (vs. GAPDH) upon 4 days of doxycycline treatment (0–12.5 mg/mL) on Tet-shDDR2 SHSY5Y cells. (**C**) Cell survival/proliferation time course assay (as measured by % confluency) of Tet-shDDR2 SHSY5Y cells treated with doxycycline (on day 0) at increasing concentrations as indicated (*n* = 6 wells/condition). (**D**) Western blot of DDR2 and phospho-tyrosine 740-DDR2 (phY740-DDR2) from Tet-shDDR2 SHSY5Y cells pretreated for 3 days with the indicated concentrations of doxycycline and left unstimulated or induced with 25 mg/mL of rat-tail type I collagen for 16 h. Protein quantification data for Western blots can be found in Supplementary Materials.

Neuroblastoma cell lines and tumors express relatively high levels of DDR2, which is significantly correlated to a worse patient survival probability [20]. Figure 1A shows the expression of DDR2 mRNA across hundreds of cancer cell lines (and non-transformed control cell lines) from the Dependency Map Portal (depmap.org, Broad Institute). Cell lines are grouped by tissue of origin/primary tumor. As it can be clearly seen from this graph, neuroblastoma cell lines—including SH-SY5Y cells—express a high level of DDR2 mRNA relative to most other cell lines in the study, which is evident both as the average/median expression and for each single peripheral nervous system cell line (except for one cell line, which indeed corresponds to the Nerve Sheath Tumor cell line HSSCH2, not neuroblastoma). The Tet-pLKO-puro shRNA system was utilized to generate shDDR2 cells, in which DDR2 knockdown can be induced by doxycycline. Western blot analysis confirmed the efficient knockdown of DDR2 expression with doxycycline concentrations ranging from 0.02–12.5 mg/mL (Figure 1B). A high expression level of DDR2 from SH-SY5Y was confirmed by Western blot analysis in the Tet-shDDR2-expressing SH-SY5Y cell system in the absence of doxycycline (Figure 1B). Doxycycline treatment efficiently induced DDR2 downregulation in Tet-shDDR2 SH-SY5Y cells (Figure 1B). Importantly, a loss of DDR2 expression did not result in any gross alterations on cellular viability, except at the highest dose tested (12.5 mg/mL), an effect likely due to unspecific toxicity (Figure 1C and Figure S1). As expected, doxycycline-mediated downregulation of DDR2 resulted in a critical impairment of collagen-dependent autophosphorylation on tyrosine 740 of DDR2, the first and indispensable signaling event triggered upon collagen/DDR2 ligation (Figure 1D). In conclusion, a novel lentiviral transduction and selection were successfully achieved to make stable SH-SY5Y cells with an inducible shRNA knockdown, with dramatic consequences in DDR2-mediated signaling.

### 3.2. Dependence of Human Neuroblastoma Phenotype on Collagen I and DDR2

**Figure 2:**
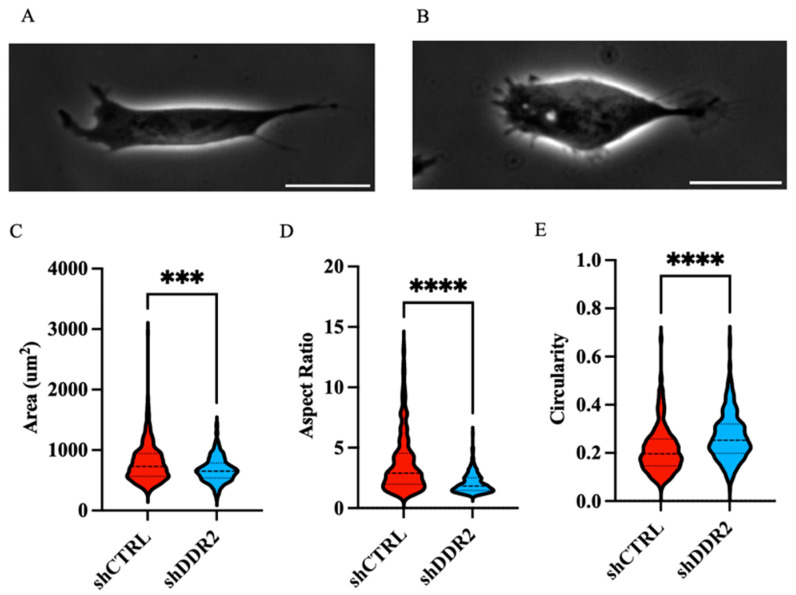
Cell morphology analysis on collagen-coated glass substrates. Representative phase-contrast images of (**A**) shCTRL and (**B**) shDDR2 cells on collagen-coated glass substrates. Quantification of (**C**) cell area (**D**) aspect ratio and (**E**) circularity. Experiments were performed in three independent experiments. Unpaired *t*-test, *** *p* < 0.001; **** *p* < 0.0001 (*n* = 228–329 cells). Data are presented as ±s.e.m. Scale bars represent 20 μm.

DDR2 alterations (overexpression, amplification, and mutations) are known to drive more aggressive phenotypes in several cancer types [39,40,41,42,43,44]. Morphological adaptations and their maintenance are a consequence of the microenvironmental influence, where a cascade of interactions can determine the cellular behaviors via signaling pathway activation and cytoskeleton arrangement [45]. Here, we measured the morphological characteristics of DDR2-expressing cells and DDR2-suppressed cells. Sitravatinib, a multi-kinase inhibitor with an affinity for DDR2 and other similar kinases, has shown effectiveness in preclinical models of high-risk neuroblastoma [46]. This drug is in phase III clinical trials as it can shift the tumor microenvironment (TME) towards an immunostimulatory state [47]. When cultured on collagen I-treated glass, shCTRL cells displayed increased areas and aspect ratios with decreased circularity compared to shDDR2 and Sitravatinib-treated cells (Figure 2 and Figure S2). Due to an increase in area and aspect ratio, shCTRL cells exhibit a more invasive, spindle-shaped cell morphology. In conclusion, higher DDR2 expression levels in shCTRL cells suggest that DDR2 plays a role in maintaining an aggressive phenotype.

### 3.3. DDR2 Silencing Altered Cell Migration on 2D Collagen Substrates

**Figure 3:**
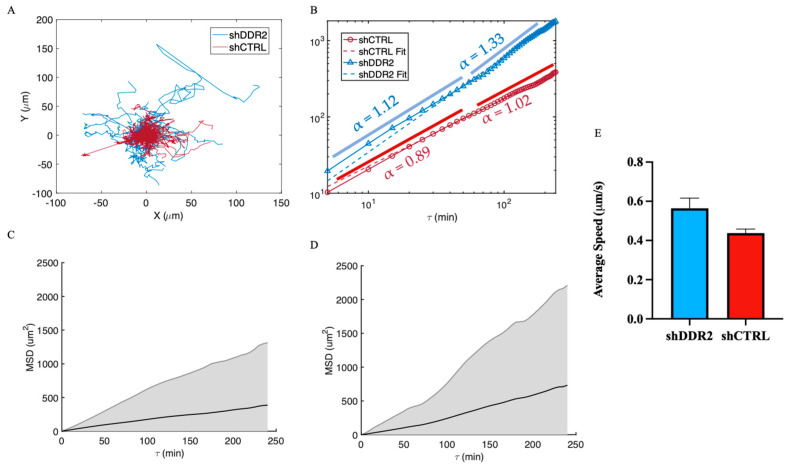
Mean square displacement of 2D single cell migration. (**A**) Individual trajectory maps of shCTRL (red) and shDDR2 (blue). (**B**) Log–log plot of MSD potted as a function of time interval for both shCTRL (red, α = 0.8838) and shDDR2 (blue, α = 1.247). Mean of the mean square displacement with shading representing the weighted standard deviation over all MSD for (**C**) shCTRL and (**D**) shDDR2 2D migrating cells. (**E**) Velocities from MSD of shCTRL and shDDR2 (*n* = 122 cells shCTRL, 55 cells shDDR2). Mann–Whitney Test, *p* = 0.0531. Data are represented as ±s.e.m.

Mean squared displacement (MSD) is often used in physics as a metric to quantify the movement of particles. The MSD represents an average squared displacement over increasing time intervals between positions of a migration trajectory. In relation to cell migration, MSD can represent the surface area explored by the cells over a time interval, which is related to the overall efficiency of migration. Cell centroids on 2D collagen-coated glass slides were tracked for 4-h time lapses, and trajectories were plotted (Figure 3A). Furthermore, a logarithmic scale of MSD was plotted for both shCTRL and shDDR2, and the exponent, or slope, α, was found to describe the migration patterns (Figure 3B). An alpha value of 0.88 describes the shCTRL data and 1.25 describes the shDDR2 data. It is important to note that at shorter time scales, shCTRL cells start in the sub-diffusive region (α= 0.89) and increase to diffusion (α = 1.02) after 150 min. However, shDDR2 begins in normal diffusion (α = 1.12) and switches to super-diffusive (α = 1.33) after 75 min. Initial dynamics of 2D cell migration typically correspond to their initial phase of particle motion. This early phase can provide insights on how cells are influenced by their initial conditions. Later time points capture the long-term average behavior of the cells. In this study, shDDR2 cells exhibited higher diffusion coefficients than shCTRl cells at both short and long time scales. To understand the overall trend that each cell line employs through 2D cell migration, the average of the MSD was plotted with standard deviations. Together, these results reveal shDDR2 exhibited higher variability, resulting in a less predictable or definitive path for these cells. Similarly, the average speed for shDDR2 cells (0.56 μm/min) is greater than (*p* = 0.053) that of the shCTRL cells (0.44 μm/min) (Figure 3C). Together, this 2D migration study suggests that downregulating DDR2 led to an increase in the migration of cells on 2D collagen-coated surfaces.

### 3.4. DDR2 Affects Cellular Protrusion Dynamics

Cell migration involves a cyclic coordination of protrusion at the cell front, adhesion of the newly protruded domain to the substrate, and pulling of the bulk of the cell towards new adhesion sites and breaking of adhesion and retraction at the cell rear. For migration, cells must elongate and retract. Filopodia, a subcellular structure at the cell front, plays a key role in the spreading and migration of cells. Thus, changes in filopodia length during the elongation and retraction stages are crucial for effective cell migration [48].

**Figure 4:**
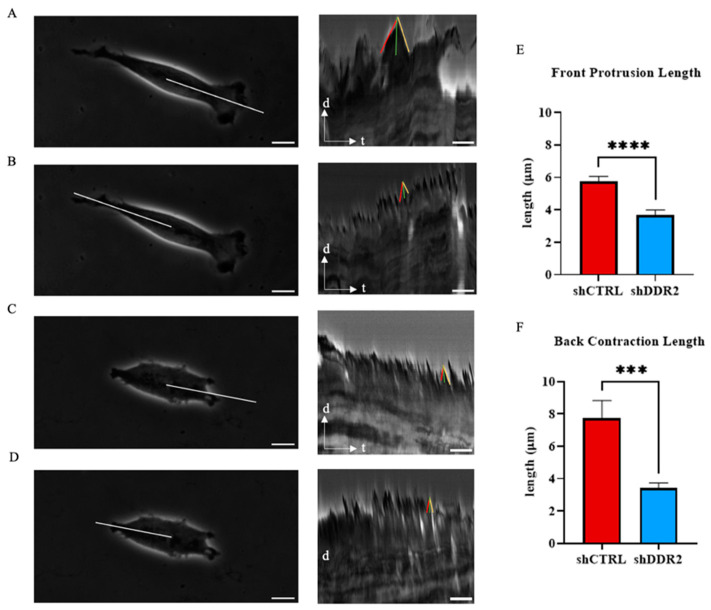
Protrusion lengths of cells on collagen-coated substrates. Representative phase-contrast image of cells on collagen-coated glass substrate with kymographic analysis of white line on shCTRL cell (**A**) front and (**B**) back and shDDR2 cells (**C**) front and (**D**) back. Kymograph-derived (**E**) front protrusive length and (**F**) back contraction length. Red line represents protrusion, yellow line represents contraction, and green line represents event length. Experiments were performed in three independent experiments. Mann–Whitney test, *** *p* < 0.001; **** *p* < 0.0001. Data are presented as ±s.e.m. Scale bars on left images represent 10 μm (**A**–**C**) and in kymographs represent 250 s (**B**–**D**).

In this work, we analyzed the front protrusion and back contraction lengths for shCTRL and shDDR2 cells. Kymographs generated from 30-min time-lapse videos on collagen-coated glass allowed the quantification of front protrusion and back contraction lengths (Figure 4A–D). The analysis revealed an increase in the front protrusion length of shCTRL and in the back contraction length compared to DDR2-downregulated cells (Figure 4E,F). These findings suggest that DDR2 suppression leads to decreased filopodia front protrusion and rear contraction elongation. These results indicate that DDR2 expression may promote cell mechanosensing and migration in vitro through filopodia extension.

### 3.5. Regulation of Cell Stiffness with DDR2 Collagen Activation

**Figure 5:**
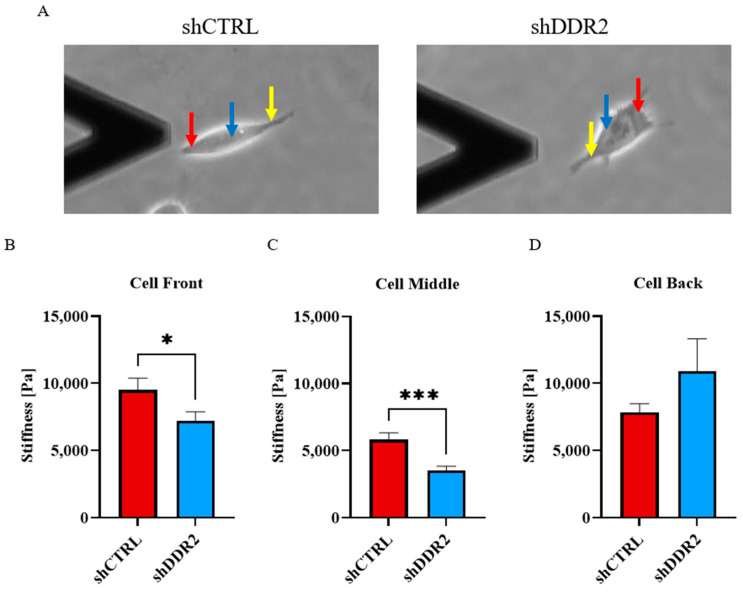
DDR2 downregulation decreases neuroblastoma cell stiffness. Young’s modulus measurements of (**A**) shCTRL and shDDR2 cells using atomic force microscopy. Indentation of (**B**) cell front (red arrow), (**C**) cell middle (blue arrow), and (**D**) cell back (yellow arrow). Young’s modulus of experiments were performed in three independent experiments. Unpaired *t*-test, * *p* < 0.05; *** *p* < 0.001 (*n* = 32–33 cells). Data are presented as ±s.e.m.

Cell stiffnesses can be related to the cytoskeletal structures within the cells, where low cell stiffness is attributed to the disorganization of cytoskeletal structures and a reduction of actin filaments [49]. To analyze the relationship between cell stiffness and DDR2 expression, we investigated the local cell stiffness using atomic force microscopy (AFM). Measurements were taken at the front, middle, and rear of cells cultured on collagen-coated glass. For the detection of the Young’s modulus, the AFM cantilever tip is pressed onto the cell surface, with the Young’s modulus defined as the ratio between the measured stress and strain. Utilizing AFM enabled us to pinpoint cell stiffnesses at the cell front, back, and center (Figure 5A). A comparison between the front and middle parts of the cell revealed significantly higher stiffnesses for shCTRL (Figure 5B,C). However, no difference was observed in the rear stiffness between shCTRL and shDDR2 cells (Figure 5D). These results indicate that downregulating DDR2 in neuroblastoma cells decreases the cell stiffness.

### 3.6. Effect of DDR2 Knockdown on Cell Traction Force

**Figure 6:**
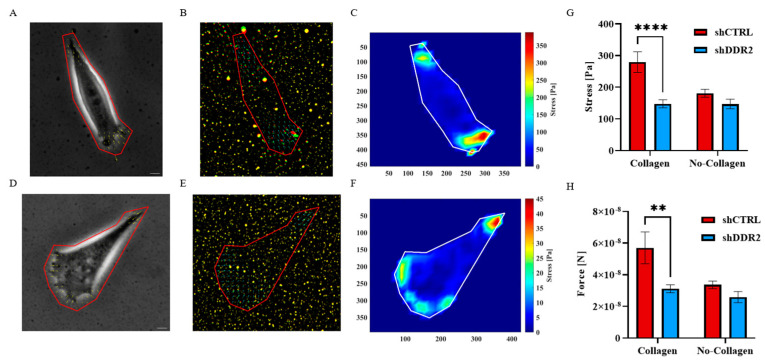
Traction force microscopy on 2 kPa collagen PAA gels. Representative images of phase contrast, force displacement field, and stress map of shCTRL (**A**–**C**) and shDDR2 (**D**–**F**) cells. Quantification of (**G**) maximum traction stress and (**H**) total force of cells on 2 kPa PAA gels (Collagen *n* = 60–65 cells, No-Collagen *n* = 45−112 cells). Experiments were performed in three independent experiments. Šídák Two-Way ANOVA, ** *p* < 0.01; **** *p* < 0.0001. Data are presented as ±s.e.m. Scale bars represent 20 μm.

To detect and interpret the biological information in the ECM, cells adhere and transduce myosin-generated traction forces through integrin-based adhesions. This process, which provokes dynamic signaling events, is termed mechanotransduction [50]. A reduction in net adhesion and migration rates, attributed to decreased binding strength and force generation at the leading edge, can lead to partial or complete loss of migration [51]. To understand the consequences of DDR2 downregulation on mechanotransduction, traction force measurements were taken on 2 kPa PAA gels for both shCTRL and shDDR2 cells, replicating the stiffness of the human brain (Figure 6A–F). Cellular traction forces have been shown to mediate mechanotransduction, cell migration, adhesion, and ECM remodeling [52]. shDDR2 cells exhibited dramatically reduced traction force compared to shCTRL. A significant difference was observed in the percentage of cells attached to the PAA gels with and without collagen for both shCTRL and shDDR2 cells (Figure S3). The absence of collagen I cross-linked onto PAA gels resulted in decreased stress and total force in shCTRL cells (Figure 6G,H). To contrast DDR2 and collagen, traction force microscopy (TFM) was performed on fibronectin-coated surfaces for both DDR2-downregulated cells and control cells. Contrary to collagen, in the presence of a different ECM protein, fibronectin, shCTRL and shDDR2 did not result in any differences to their traction force (Figure S4). This data highlights the specificity of shCTRL cells for collagen. In summary, these experiments suggest that DDR2-downregulated neuroblastoma cells exhibit altered mechanotransduction activity when plated on collagen-I cross-linked PAA gels.

## 4. Discussions

Cancer invasion is inherently coupled to cell mechanics and ECM properties [53,54,55]. These cellular mechanics, intertwined with matrix mechanics, allow cells to remodel the surrounding matrix environment by exerting forces on it. DDR2 has been shown to play a role in the growth and metastasis of epithelial and mesenchymal cancers [56,57,58,59,60,61,62]. However, the role DDR2 plays in the metastasis of neuroblastoma remains ill-defined.

In the present study, we provide the first study, to our knowledge, utilizing shRNA technology on SH-SY5Y cells to elucidate the role of DDR2 in mediating neuroblastoma cell mechanics and migration. One of the most prominent characteristics of cancer cells is their ability to proliferate constantly. We found that downregulating DDR2 was a critical regulator of neuroblastoma cell maintenance. Most receptor tyrosine kinases (RTKs) bind to soluble growth factors and mediate various cellular responses, including proliferation, differentiation, migration, and survival [63,64]. Various studies have demonstrated that DDR2 regulates cellular proliferation in embryonic cells [65], breast cancer cells [66], endochondral cells [67], and squamous cells [68]. Therefore, it is not surprising that DDR2 is also essential for neuroblastoma growth.

Inhibiting DDR2 has been shown to decrease the migration of melanoma [26], fibroblasts [69], breast cancer [22], and lung cancer [70]. Previous migration studies on DDR2 have been conducted through long-term (6–24 h) migration assays by measuring the amount of cells invaded from populated regions into a previously empty region [25,26]. However, both cell migration and cellular proliferation contribute to these cell invasion studies, making it hard to decouple the singular invasion effect. In this work, we directly measured the effects of downregulating DDR2 expression on the 2D migration of neuroblastoma by tracking individual cells’ MSD, which has been utilized as a tool to gain insight on the migratory mode [71,72,73]. Notably, MSD for both shCTRL and shDDR2 exhibits a non-linear increase with τ, indicating anomalous dynamics of their migration. At a shorter time scale (τ<60  min), the migration of both cell lines can be modeled as normal Brownian motion, with an anomalous exponent α approximately equal to 1. At a longer time scale, the α value of the shDDR2 cell line increased to 1.3, suggesting that knocking down DDR2 shifts the cell migration mode from diffusive mode to super-diffusive mode as time increases. Although our results show no statistical significance (*p* = 0.053), there is an increased trend for the average migratory speed of the DDR2-downregulated cells. The biphasic relationship between cell migration and adhesion has been well studied [74,75]. DDR2 depletion has been shown to decrease the cell focal adhesion size [76] which consequently alters the migration of cells [77]. With the knowledge that DDR2 autophosphorylation begins after binding to collagen, it is hypothesized that this lack of DDR2 binding to collagen in shDDR2 cells causes an increase to the MSD anomalous exponent into the super-diffusive region.

shDDR2 SH-SY5Y cells also reduced their cell area and aspect ratios, consistent with other reports [25]. In addition to morphology changes, the net traction forces, frontal protrusion length, and stiffnesses decreased for the DDR2-downregulated cells. These results suggest that DDR2 could modulate a mesenchymal phenotype [78]. Some reports associate decreased cell stiffness with cancer invasiveness [79,80]. However, cancer cell invasion is a complex process that cannot be predicted by a single cell parameter alone [81]. As a first step of mesenchymal cell migration, cells develop a leading edge that mechanically couples to the extracellular tissue, which is followed by cell contraction, gradual sliding of the cell rear, and translocation of the cell body [82]. The filopodia at the leading edge positively controls mesenchymal migration directionality and persistence [83,84]. The data presented in this work shows the coupling of increasing protrusion and retraction lengths which complements the increased aspect ratios of the cellular morphologies. Together, our data suggests that DDR2 affects the ability of these cells to elongate this filopodia and continue in a metastatic cascade. Cell stiffness can often be attributed to the organization and bundling of actin filaments [85] which is likely due to the increase in protrusion lengths for the neuroblastoma cells studied here. Bayer et al. [76] found decreased cell spreading coupled with reduced traction forces on DDR2 depleted cancer-associated fibroblasts (CAFs), which agrees with our findings in SH-SY5Y neuroblastoma cells. Additionally, they found that DDR2 was required for full activation of collagen binding to β1-containing integrins. It is plausible that DDR2 modulates integrin-mediating mechanics to collagen substrates in this neuroblastoma cell line. In corroboration with this, we show that it is not migration speed alone that controls the metastatic ability of SH-SY5Y cells, but rather an interplay between the proliferation, protrusion dynamics, traction forces, and cell stiffness.

In summary, we provide the first study, to our knowledge, on the possible mechanical changes generated by the collagen-binding receptor, DDR2, in the neuroblastoma cell line, SH-SY5Y, for controlling the mesenchymal phenotype. We also show that DDR2 is a critical pathway to control aggressive behavior that forms in neuroblastoma and that facilitates tumor cell invasion. It is important to note that these changes may vary between cell lines. As such, DDR2 could represent an important therapeutic target and have a significant impact on cancer progression. Our results also shed light on the action of DDR2 in neuroblastoma cells, which may contribute to migration mechanics and cell–cell and cell–ECM interactions.

## 5. Conclusions

In this study, we used a novel neuroblastoma cell line with normal and downregulated DDR2 levels to conduct a study on the cellular mechanics of DDR2 upon activation of collagen I. We employed various parameters to verify the robustness of our results. The combined results obtained for control and DDR2-downregulated cells suggest that collagen activation of DDR2 tyrosine kinase is required for normal neuroblastoma growth and spreading. Similarly, the neuroblastoma cells with downregulated DDR2 exhibited significantly decreased cell stiffnesses, protrusion lengths, and, consequently, lower traction forces compared to the control neuroblastoma cells. However, a dynamic balance between these variables is necessary, as an increase in cell stiffness causes a decrease in the MSD. These data signify the importance of DDR2 and collagen signaling by elucidating the mechanisms involved in the mesenchymal neuroblastoma cell line migration mechanics. Our results provide insight into the mechanics of collagen-binding receptor DDR2 as a critical pathway for controlling aggressive neuroblastoma cells.

## Data Availability

The original contributions presented in the study are included in the article/Supplementary Material, further inquiries can be directed to the corresponding authors.

## Supplementary Materials

The following supporting information can be downloaded at: https://www.mdpi.com/article/10.3390/life14101260/s1. Supplementary Information S1: Figure S1. shiDDR2 confluency with treatment of doxycycline. Representative bright field images of shDDR2 cell line with untreated, 0.02 mg/mL–15 mg/mL of doxycycline. Figure S2. Morphology of cells attached to collagen coated glass substrates morphology. Quantification of (A) cell area (B) cell aspect ratio and (C) cell circularity of control, control cells treated with 0.05% DMSO, and Sitravatinib treated cell. Experiments performed in three independent experiments (*n* = 184–329 cells). Un-paired t-test, *p* < 0.05. Data are presented as ±s.e.m. Scale bars represent 20 μm. Figure S3. Percentage of cells attached onto 2kPa PAA gel. Percentage of cells that remain attached to collagen coated PAA gel from before and after vigorous rinsing with DPBS. Experiments performed in three independent experiments. Tukey 2Way ANOVA, *p* < 0.05. Data are presented as ±s.e.m. Figure S4. Traction force microscopy on fibronectin coated 2kPa PAA Gel. (A) total force and (B) maximum traction stress (*n* = 22–27 cells). Experiments performed in three independent experiments. Unpaired *t*-test, *p* < 0.05. Data are presented as ±s.e.m. Supplementary Information S2: Protein quantification relevant to Figure 1B,D.
