# Supplementary material for: Investigation of Cell Mechanics and Migration on DDR2-Expressing Neuroblastoma Cell Line
Source: Life (Basel). 2024 Oct 2;14(10):1260. doi: 10.3390/life14101260 (PMC11509142; doi:10.3390/life14101260)
Supplement: Supplementary file 1 [file life-14-01260-s001.zip › Supplementary Information S1.pdf]

Supplemental Information S1

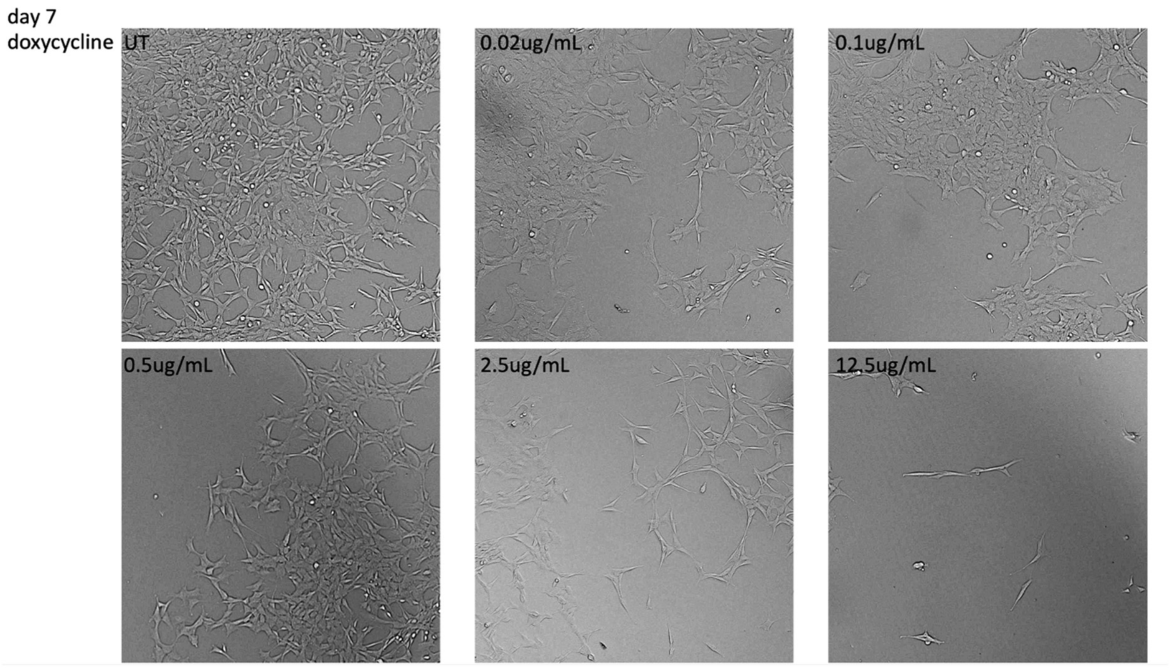

Figure S1. shiDDR2 confluency with treatment of doxycycline. Representative bright field images of shDDR2 cell line with untreated, 0.02 mg/mL – 15 mg/mL of doxycycline.

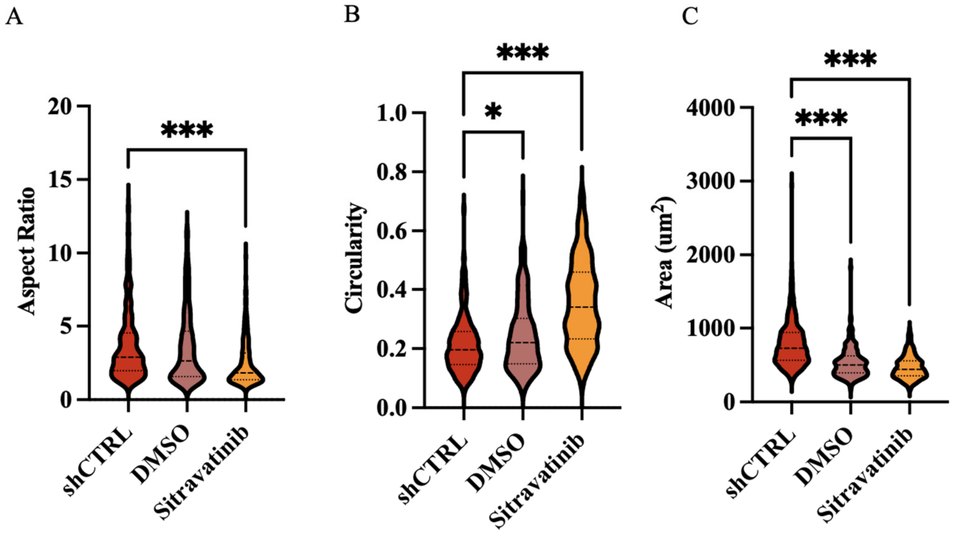

Figure S2. Morphology of cells attached to collagen coated glass substrates morphology. Quantification of A) cell area B) cell aspect ratio and C) cell circularity of control, control cells treated with 0.05% DMSO, and Sitravatinib treated cell. Experiments performed in three independent experiments (n=184-329 cells). Un-paired t-test, $p < 0.05$. Data are presented as ± s.e.m. Scale bars represent 20 μm.

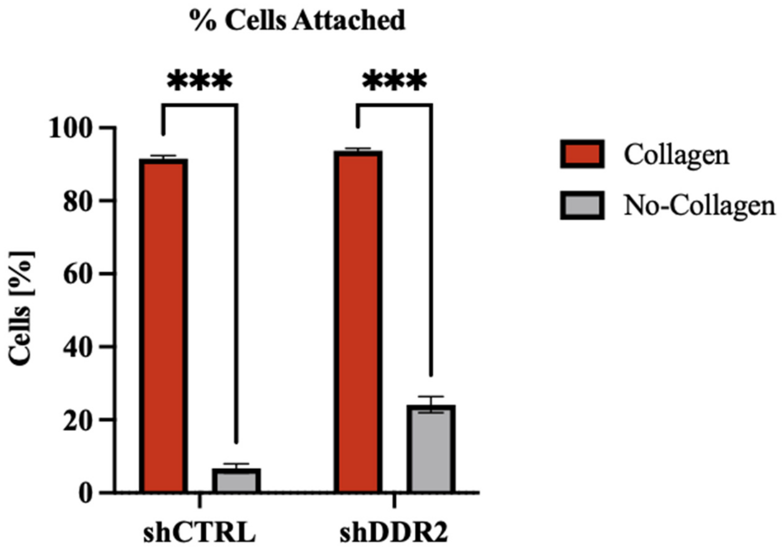

Figure S3. Percentage of cells attached onto 2kPa PAA gel. Percentage of cells that remain attached to collagen coated PAA gel from before and after vigorous rinsing with DPBS. Experiments performed in three independent experiments. Tukey 2Way ANOVA, $P<0.05$. Data are presented as ± s.e.m.

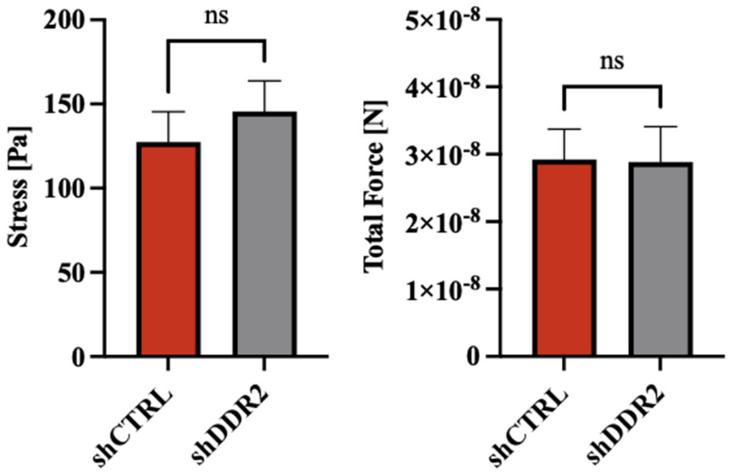

Figure S4. Traction force microscopy on fibronectin coated 2kPa PAA Gel. A) total force and B) maximum traction stress (n= 22 - 27 cells). Experiments performed in three independent experiments. Unpaired t-test, $P<0.05$. Data are presented as ± s.e.m.
